# Assessing the social impacts of the COVID-19 crisis using phone helplines. The case of the Balearic Islands, Spain

**DOI:** 10.3389/fpubh.2024.1270906

**Published:** 2024-03-13

**Authors:** Maria Ramos Monserrat, Jeronia Ramón Molinas, Marta Fuster Truyol, Aina Bonet Manresa, Trinidad Planas Juan, Juan José Montaño Moreno, María de los Ángeles Pérez Martín, Patricia Ruíz Armengol, Almudena Personat Labrador, Carlota María Lamilla Buades, Verónica María Carrión García, Miguel Salvá Garví, Catalina Nuñez Jiménez, Elena Cabeza Irigoyen

**Affiliations:** ^1^Balearic Islands Public Health Department, Palma, Spain; ^2^Balearic Islands Health Research Institute (IdISBa), Palma de Mallorca, Spain; ^3^University of Balearic Islands, Palma de Mallorca, Spain; ^4^Balearic Health Services, Palma, Spain

**Keywords:** social determinants of health, COVID-19, helplines, inequalities, territory

## Abstract

**Background:**

Crises and health policies to tackle them can increase health inequalities. We explored the scope and usefulness of helplines set up during the COVID-19 crisis and characterised the vulnerability of their users. This study explored the geographic and socioeconomic effects of the telephone helplines set up by the Balearic Islands Government and aimed to characterise the vulnerability of their users.

**Methods:**

Telephonic survey combined with a geographical analysis of a sample of calls made between 15th of March and 30th of June of 2020 to five helplines: COVID-19 general information; psychological, social (minimum vital income), labour (temporary employment regulation), and housing (rental assistance) helps. The questionnaire included sociodemographic and housing characteristics, type of problem, and if it was solved or not. We used multinomial regression to explore factors associated with having solved the problem. We calculated the standardised rate of calls by municipality using Chi-squared and *z*-test to test differences.

**Results:**

1,321 interviews from 2,678 selected (231 excluded, 608 untraceable, and 518 refusals). 63.8% of women, 48.7% were born in another country. They had no internet at home in 3.1%, only on the phone in 17.3%. The 23.5% had no income at home. The Problem was solved in 25.4%, and partly in 30.9%. Factors associated with not solving the problem were not having income at home (*p* = 0.021), labour (*p* = 0.008), economic (*p* = 0.000) or housing (*p* = 0.000) problems. People from 55 of 67 municipalities did at least one call. The highest rates of calls were from coastal tourist municipalities.

**Conclusion:**

Helplines reached most of the territory of the Balearic Islands and were used mainly in tourist municipalities. It probably has not been helpful for families with more significant deprivation. Digital inequalities have emerged.

## Highlights

- Helplines probably have not been helpful for the most vulnerable families in the COVID-19 crisis.
- Digital inequalities have emerged during the COVID19 pandemic that should be addressed without waiting for a new crisis.

## Introduction

In the COVID-19 crisis, some of the public health measures adopted, namely the lockdown policies, have exacerbated health inequalities, as happened or occurred in other pandemics (1, 2). Therefore, COVID-19 has been qualified as a “syndemic,” as it interacts with and exacerbates the consequences of the social determinants of health (3).

Since the start of the COVID-19 pandemic, many efforts have been made to identify population groups that were more vulnerable to severe COVID-19 infection or death. However, the identification and support of more vulnerable groups in terms of social and economic consequences (2, 4) should have been equally important in terms of resourcing and advocacy, because their vulnerability was exacerbated by the pandemic (5, 6).

There were many uncertainties in the COVID-19 pandemic, and the Geographic Information Systems (GIS) was essential to understand and predict the disease’s evolution, track human movements, detect and help vulnerable groups, and formulate and examine health policy interventions (7).

The Spanish Government set up different actions to reduce the social and economic consequences of COVID-19, the so-called Social Shield. These actions included ensuring a minimum vital income and enforcing measures of temporary employment regulation (8). Concurrently, the Balearic Islands Government launched phone helplines to offer information about COVID-19, to provide psychological support, and help to complete applications for the social assistance measures mentioned above, as well as a website compiling all the information related to COVID-19.^1^

This study explored the geographic and socioeconomic effects of the telephone helplines set up by the Balearic Islands Government and aimed to characterise the vulnerability of their users.

## Methods

### Design

Descriptive study that combined a telephonic survey and a geographical analysis.

### Population of study

Residents in the Balearic Islands, Spain (1,171,543 inhabitants in 2020, 50.1% women). The Balearic Islands inhabited are four: Mallorca (77.9% of the Balearic Islands’ population), Menorca (8.2%), Ibiza (12.9%), and Formentera (1%). Palma is the capital city (36.1% of the Balearic Islands’ population) (9).

### Sampling

We selected a non-probabilistic convenience sample of telephone numbers from Balearic Islands residents who have called during the period of strict lockdown in Spain (between the 15th of March and the 30th of June 2020). We included the following helplines: COVID-19 general information; psychological help; social help, for minimum vital income; labour help, for applying to temporary employment regulation (ERTE, in Spanish) and housing rental help. Furthermore, we excluded the lines addressed to women victims of gender based violence (GBV) and minors to avoid putting them in danger. The criteria for selecting the sample were numbers that call to more lines, numbers that call more times to each line, and those that call for social and housing help. We assumed that we would include the most vulnerable people in this way. We made as many calls as we could with the resources we had. We included both mobile and landline numbers.

### Procedures

A team of trained health professionals called the numbers selected and interviewed the respondents’ after asking for informed consent. We made three attempts on different days and hours. The only exclusion criterion for the interview was that the phone number corresponded to a municipal service or business consultancy instead of a citizen. Interviews took place between the 1st of August 2020 and the 31st of March 2021, and they were recorded.

### Instruments

The research team drew up the questionnaire used (Annex 1).

### Variables

(1) Sociodemographic characteristics: age, sex, country of birth, and municipality or neighbourhood of residence; number, age, and labour situation of persons living at home; (2) housing conditions: m2 of the house; access or not to exterior spaces at home (balcony, terrace, and garden); access to the internet in the house or on the mobile phone, and (3) reason for the call: type of problem or problems they called for, and if the problem they have called for was solved or not (yes, no, partly yes, and partly no).

### Analysis

#### Survey

We performed a descriptive statistical analysis with relative frequencies, a bivariate analysis with a Chi-square (*X*^2^) test to explore factors associated with the variable if the problem they have called for was solved, and multinomial regression, as the dependent variable had three categories. The software used was SPSS 17.0.

#### Geographic analysis

We allocated the calls according to the municipality and neighbourhood (only for Palma) on a map. Then, we calculated the rate of calls by municipality and neighbourhood by population, and we standardised them based on the overall rate for the Balearic Islands. We applied a global *X*^2^ test with a continuity correction for island, municipality, and neighbourhood to analyse the differences between the observed and the expected calls based on the total number of registered calls and the population sizes. Next, we applied an individual *z*-test with a continuity correction (10), controlling the alpha error through the Bonferroni correction for each island, municipality, and neighbourhood. Finally, we built a Geographical Information System (GIS) combining the information about the calls by municipality or neighbourhood (number, rate, and standardised rates) with socioeconomic information by censual section: income rates and Gini index by census section (11), and we compared the patterns visually. The software programmes used were ArcGis and QGis.

## Results

Between 15 March 2020 and 30 June 2020, 42,532 telephone numbers called to the helplines: 11.1% for COVID-19 general information, 2.0% for psychological help, 20.5% for social help, 51.4% for labour help and 24.8% for housing rental help. A sample of 2,678 numbers was selected (6.4%) (Supplementary Table 1). Finally, 1,321 interviews were performed, as 231 cases were excluded, 608 were untraceable and 518 were refusals to participate.

The sociodemographic and housing conditions of persons interviewed are shown in Table 1. Two out of three were women, with an average age of 42.25 (SD: 11.141). Half were born in another country (46 different countries). The most frequent countries of origin were Spain (667 cases), followed by Argentina (113 cases), Colombia (108 cases), Ecuador (50 cases), Morocco (36 cases) and Italy (36 cases). They lived with an average of 2.77 persons (SD: 1.423), more frequently with children (43.7%) than with aged people (10.5%). Houses had <80 m^2^ in 54.7% of cases, with an average of co-habitants of 2.77 (SD: 1.423), without any exterior space in 16.4% of cases and access to the internet in 3.9%, or only on the mobile phone in 17.3%. The problems they called for were mainly economic, followed by labour and housing. 23.5% of interviewers declared that they had no income at home, and in 21.3% of cases, there was someone in the house in ERTE. Only 7% of the people interviewed said they had a psychological problem.

**Table 1:** Description of persons interviewed (*N* = 1,321).

Variable	Categories	Number	Percentage
Sex	Women	835	62.8
	Men	482	36.3
	Unknown	12	0.9
Age	<24	44	3.3
	25–34	302	22.7
	35–44	453	34.1
	45–54	334	25.1
	55–64	139	10.5
	≥65	43	3.2
	Unknown	14	1.1
Island of residence	Mallorca	1,040	78.3
	Menorca	58	4.4
	Eivissa	192	14.4
	Formentera	26	2.0
	Unknown	13	1.0
Place of birth	Balearic Islands	369	27.8
	Another Spanish region	299	22.5
	Another country	647	48.7
	Unknown	14	1.1
Continent of origin	Europe	809	60.9
	Africa	64	4.8
	America	424	31.9
	Asia + Oceania	13	1
<18 years old persons at home	No	742	55.8
	Yes	581	43.7
	Unknown	6	0.5
≥65 years old persons at home	No	1,184	89.1
	Yes	139	10.5
	Unknown	6	0.5
m^2^ house	< 40 m^2^	88	6.6
	41–60 m^2^	240	18.1
	61–80 m^2^	379	28.5
	81–100 m^2^	421	31.7
	101–120 m^2^	110	8.3
	>120 m^2^	51	3.8
	Unknown	40	3.0
Balcony	Yes	579	43.6
Terrace	Yes	469	35.3
Garden	Yes	199	15.0
Internet	Yes, at home	1,045	78.6
	Yes, on the phone	232	17.5
	No	41	3.1
	Unknown	11	0.8
Was the problem they called for solved?	No	582	43.8
	Yes	337	25.4
	Partly yes, partly no	410	30.9
Type of problem	Labour	728	54.8
	Psychologic	93	7.0
	Economic	987	74.3
	Housing	669	50.3
	Another	171	12.9
No income at home	No	1,008	75.8
	Yes	312	23.5
	Unknown	9	0.7

Issues leading the call were solved in 25.4% of cases and partly solved in 30.9%. The factors associated with not solving the problem were in the unadjusted analysis: having no income at home (*p* < 0.001), having no internet (*p* < 0.05), having children at home (*p* < 0.01) and having no one in ERTE (*p* < 0.05). After the multinomial regression, factors associated with not solving the problem were having no income at home (*p* < 0.05) or if the problem was of labour (*p* < 0.005), economic (*p* < 0.001) or housing (*p* < 0.001) (Table 2).

**Table 2:** Multinomial regression of factors associated with not solving the problem (No, as reference).

To solve the problem	Variables	*B*	Sig.	Exp (B)	CI^*^ inf.	CI sup.
Partly yes, partly no	Labour	0.480	0.000	1.616	1.242	2.104
	Economic	0.659	0.000	1.934	1.384	2.702
	Housing	0.299	0.025	1.349	1.038	1.753
	No income at home	−0.344	0.026	0.709	0.524	0.960
	Garden	0.407	0.029	1.502	1.042	2.167
Yes	Labour	−0.384	0.008	0.681	0.513	0.903
	Economic	−0.677	0.000	0.508	0.376	0.686
	Housing	−0.718	0.000	0.488	0.366	0.649
	No income at home	−0.411	0.021	0.663	0.468	0.940
	Garden	0.340	0.089	1.405	0.949	2.080

^*^CI, Confidence interval at 95%.

Regarding geographical distribution, a 78.3% of calls were from Mallorca, 4.4% from Menorca, 14.4% from Ibiza and 2% from Formentera. The number of calls was higher than expected in Formentera (*p* < 0.05). Instead, it was significantly lower than expected in Menorca (*p* < 0.05).

**Figure 1:**
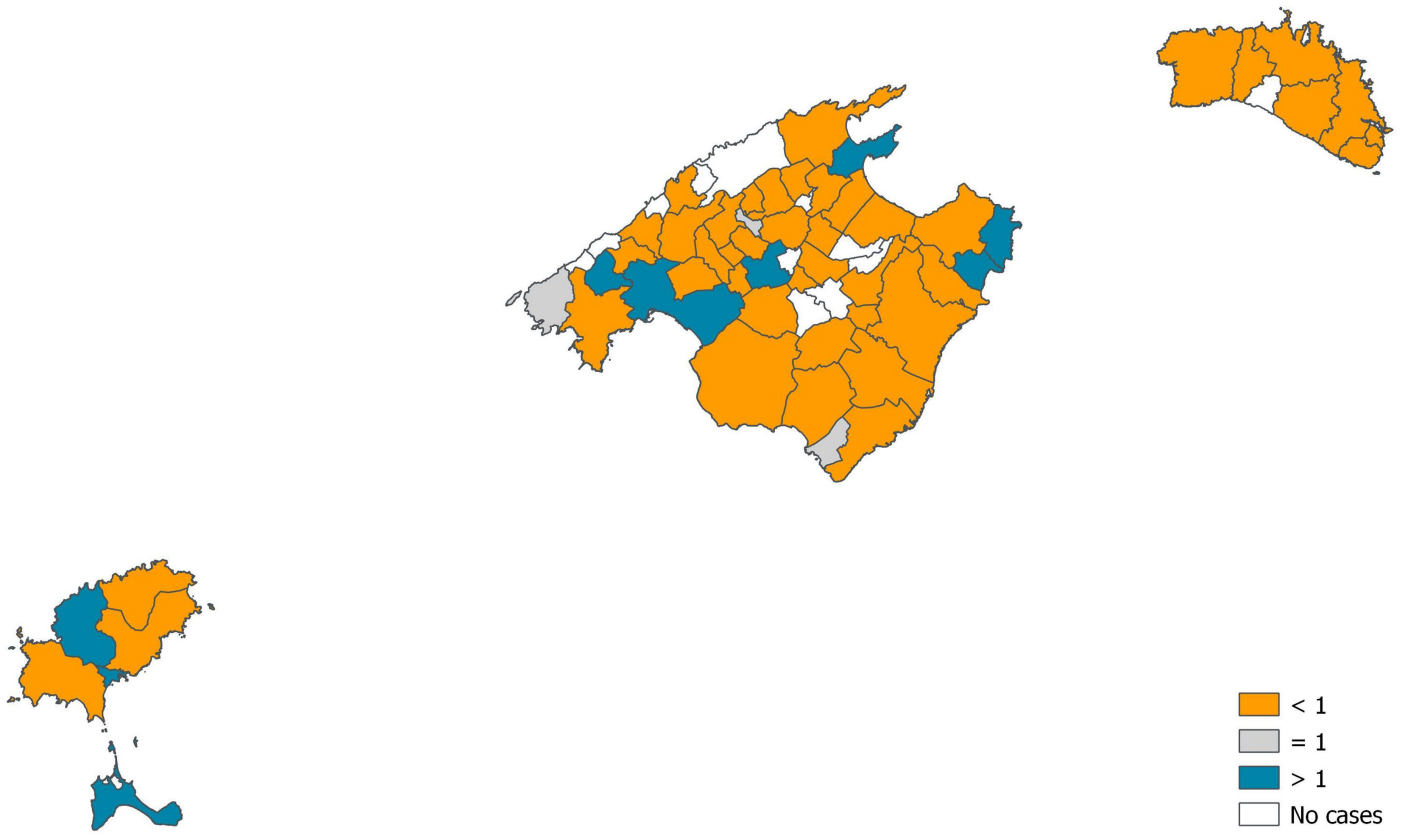
Standardised rates of calls by municipalities.

**Figure 2:**
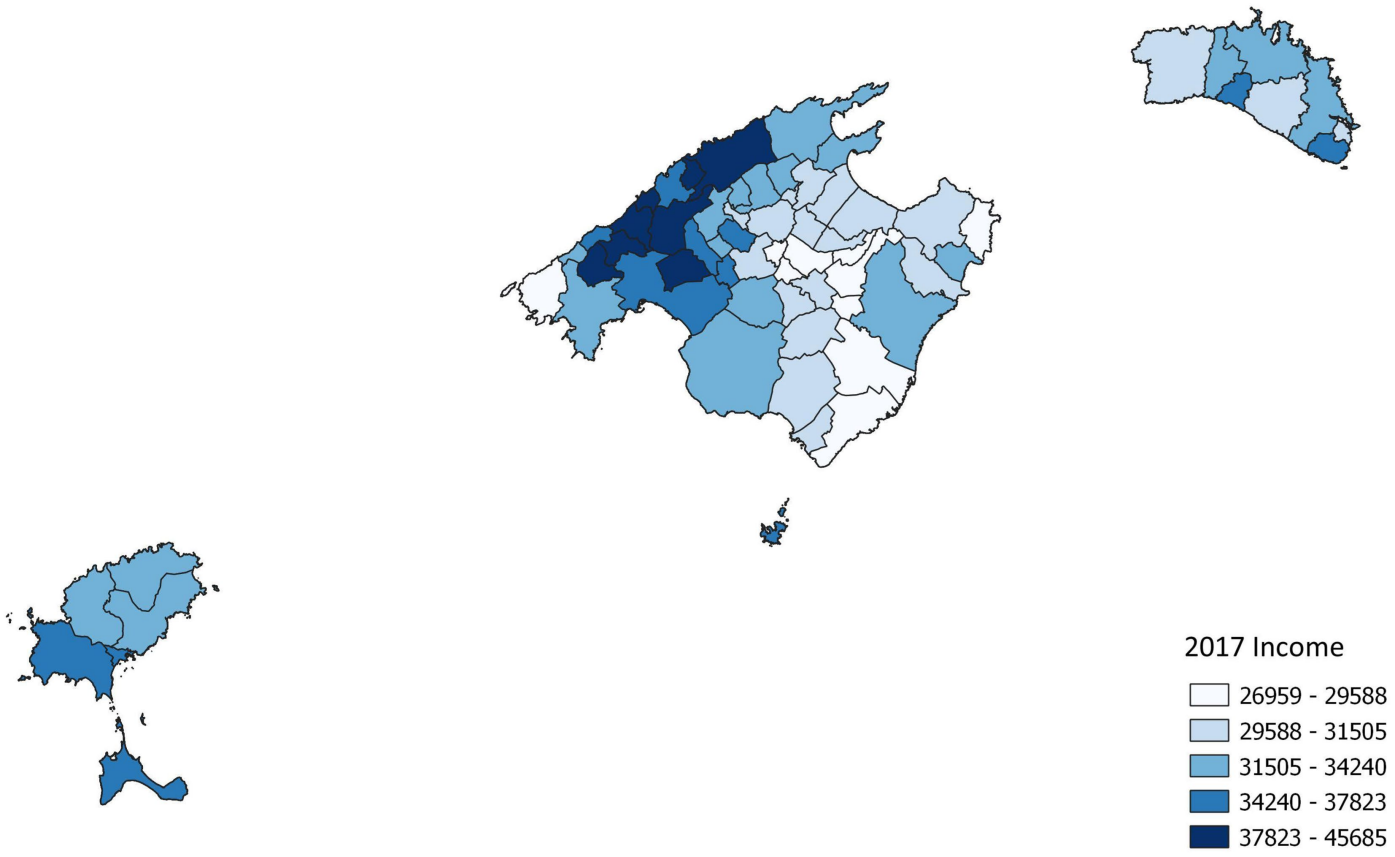
Income rates by municipalities.

At least one call was made from 55 municipalities and none from 12 (11 of Mallorca and one of Menorca). Nearly, a half of the calls (46.4%) were made from Palma city. After standardisation of call rates, we observed that the municipalities with lower calls or no calls were municipalities with low population and high income in the Tramuntana mountain range or with lower income in the interior of the island of Mallorca (Figure 1). Instead, the municipalities with the highest call rates (Figure 2) were tourist municipalities on the coast or near Palma. There were significant differences between the observed calls and the expected calls by each municipality (*p* < 0.005). Only six municipalities presented rates significantly over expected (*p* < 0.05), and only one lower than expected (*p* < 0.05), apart from the 12 without calls.

In Palma city, there were calls from 71 of 88 neighbourhoods. The neighbourhoods with rates under the expected number of calls or without calls were sparsely populated and in a situation of extreme wealth or poverty. Whilst neighbourhoods with the highest rates of calls were underprivileged and populated. There were significant differences between the observed calls and the expected calls by neighbourhood (*p* < 0.005). Only five neighbourhoods presented rates significantly higher than expected (*p* < 0.05).

When comparing standardised calls with income rates and the Gini index, we did not observe clear patterns for municipalities or neighbourhoods in Palma (Supplementary Figures 1–4).

## Discussion

We have observed that the phone helplines covered all islands and most municipalities. However, there were significant differences in their use, being higher in touristic areas. It is coherent with the economy of the Balearic Islands, based on tourism. The main reported problems were economic, labour and housing, all three interconnected and related to the interruption of tourism activities, mainly bars, restaurants and hotels.

Users of the lines were working-age people, mainly women and immigrants, reinforcing the vulnerability of immigrant people, especially women (12) working in the domestic environment (13). The absence of older people both among the persons interviewed and among the people who live with them, which we attribute to the fact that they have a pension. Our results support the idea that the social consequences of the COVID-19 crisis are associated with young age, female gender and poor economic conditions (14).

Psychological helplines were used less than expected, despite, lockdowns negatively impacting mental Health and economic difficulties are significant stressors in these situations (15). Our results suggest that offering and announcing a psychological helpline is not enough to help the people with psychological distress during a global health crisis like COVID-19, especially when the visits to the health centres are discouraged to prevent the spreading of the disease. Indeed, the stressors are related to gender, economic difficulties, worry about work, lack of information, trust in the institutional response and fear of infection (14–18).

We have identified a non-negligible percentage of people who declared no income, and this circumstance was associated with not having solved the problem through the helpline. Therefore, we hypothesise that the helplines could not help the families that need them most, that is, the families with severe financial difficulties emerge as the most vulnerable (19). We believe that a combined approach to this population group through community networks and agents (20) from day 1 of the crisis could have mitigated the social consequences of COVID-19. In the Balearic Islands, we have done it as a strategy to reduce the cases. However, we discovered that these families had difficulties maintaining lockdown conditions due to housing and employment issues.

At the same time, there is also a non-negligible percentage of people without internet at home, having it only on their phone. In this pandemic, the importance of digital inequalities has emerged as a new public health challenge (21). Smartphones have proven to be a valuable tool for searching for health information, social support between peers (4, 13) and rapid assessment of people’s needs (22) or mental health state (16). Nevertheless, we have seen in this study that smartphones are insufficient for applying for official help. Beaunoyer and colleagues have proposed strategies for reducing digital inequalities, targeting both individuals’ access and use of technologies and the messages’ quality, understandability, and acceptability (21). In the Balearic Islands, social workers have been overwhelmed trying to help people apply for official help, and the Government provided laptops to schoolchildren without them. Nevertheless, there is much work to do to reduce digital inequities in order to prepare for future crises through the joint work of Technology, Education and Social Services departments with municipalities.

### Weaknesses and strengths of the study

We designed the study in April–May 2020, the interviews started in September 2020, and we presented the results in the second half of 2021. This time span is undoubtedly too long and has been useless for this crisis, although it can teach us things for the future. We suggest designing a global strategy to evaluate helplines, using rapid online surveys (16, 22), probably immediately after the call, as commercial companies do.

One of the problems we have had is that we expected that the phone companies would provide us with the geographical location of all the calls. Finally, this was not possible, so we had to work with a sample asking for this information during the interviews. The questionnaire included only the municipality and neighbourhood, as we were afraid that people would not feel comfortable giving us their address. However, we verified that people had no problem giving us their address during the fieldwork. Addresses would allow us to do a more accurate geographic analysis, especially to compare the call rates with economic layers. We believe that the lack of visual correlation of call rates with socioeconomic information is due to the imprecision of the unit used for call rates (municipality or neighbourhood) as the unit for socioeconomic information was the census section. Therefore, we have suffered the modifiable area unit problem or ecological fallacy (23).

We believe that the combination of methodologies has been a wise choice, especially the inclusion of geographic analysis to evaluate a public health measure as the development of different helplines to mitigate the social and economic effects of a health crisis such as COVID-19. We have also used qualitative methods (24) to investigate the problems for which people called into these lines.

As far as we know, this is the first study that aims to evaluate the geographical and social effectiveness of different helplines during a health crisis. Other authors have explored the scope and usefulness but only of psychological helplines (25, 26). Other authors had used helpline calls during the COVID-19 crisis as a proxy for the mental health status of the population, observing that the number of calls increased when the restrictive measures were implemented and decreased when such measures were revoked (27).

COVID-19 has changed the world and provided an opportunity to improve it (28), although how tourism is coming back to the Balearic Islands makes us doubt it. For future pandemics, as we know that “those most vulnerable will be the hardest hit” (29), the core idea that “this size does not fit all” (30) should be applied from the beginning. The collection of socioeconomic data of cases could aid (31) but probably it will not be enough. It is necessary to be closer to the people that suffer, and primary care professionals, social workers and mental health specialists are well positioned to do it. Significant coordination between them would be convenient for a future crisis (32).

## Conclusion

The helplines set up by the Balearic Islands to mitigate the social and economic consequences of COVID-19 arrived at all islands, and most municipalities, but they have probably been no help for the most deprived families. Migrants, women, workers in the tourism sector and especially families without any income were the most vulnerable groups. Digital inequalities have emerged, and they should be addressed without waiting for a new crisis. At the same time, it seems crucial to deal with collaborative efforts between public health, primary health care, social work, and mental health sectors. The development of a global strategy for evaluating the helplines using rapid online surveys could also identify the most vulnerable groups affected by the sanitary crisis.

## Data availability statement

The raw data supporting the conclusions of this article will be made available by the authors, without undue reservation.

## Ethics statement

The studies involving humans were approved by Balearic Islands Ethics Committee. The studies were conducted in accordance with the local legislation and institutional requirements. The participants provided their written informed consent to participate in this study.

## Author contributions

MR: Conceptualization, Data curation, Formal Analysis, Investigation, Project administration, Validation, Writing – original draft. JR: Conceptualization, Methodology, Supervision, Visualization, Writing – review & editing. MF: Data curation, Formal Analysis, Investigation, Methodology, Visualization, Writing – review & editing. AB: Data curation, Investigation, Validation, Writing – review & editing. TP: Investigation, Writing – review & editing. JM: Formal Analysis, Methodology, Writing – review & editing. MP: Investigation, Writing – review & editing. PR: Investigation, Writing – review & editing. AP: Investigation, Writing – review & editing. CL: Investigation, Writing – review & editing. VC: Investigation, Writing – review & editing. MS: Data curation, Methodology, Validation, Writing – review & editing. CN: Investigation, Writing – review & editing. EC: Conceptualization, Methodology, Supervision, Writing – review & editing.

## Footnotes

^1^ www.coronavirus.caib.es
