# Supplementary material for: Assessing the social impacts of the COVID-19 crisis using phone helplines. The case of the Balearic Islands, Spain
Source: Front Public Health. 2024 Mar 13;12:1270906. doi: 10.3389/fpubh.2024.1270906 (PMC10976841; doi:10.3389/fpubh.2024.1270906)
Supplement: Supplementary file 1 [file Table_1.docx]

Supplementary Table 1. Universe and sample of helplines used in the study

| Total | Phone numbers | | Calls | |
| --- | --- | --- | --- | --- |
|  | Num. | % | Num. | % |
| COVID19 general information | 4,737 | 11.1 | 6830 | 5.4 |
| Psychological help | 867 | 2.0 | 1,259 | 1.0 |
| Social help | 8,706 | 20.5 | 16,352 | 13.0 |
| Labour help | 21,882 | 51.4 | 54,237 | 43.1 |
| Housing help | 10,552 | 24.8 | 47,032 | 37.4 |
| Total | 42,532 | 109.9 | 125,71 | 100.0 |
| Sample | Phone numbers | | Calls | |
|  | Num. | % | Num. | % |
| COVID19 general information | 55 | 2.0 | 77 | 0.7 |
| Psychological help | 16 | 0.6 | 29 | 0.3 |
| Social help | 832 | 31.1 | 1,601 | 14.9 |
| Labour help | 930 | 34.7 | 2,466 | 23.0 |
| Housing help | 1,001 | 37.4 | 6,533 | 61.0 |
| Total | 2,678 | 105,8 | 10,706 | 99.9 |
