# Supplementary material for: Assessing the social impacts of the COVID-19 crisis using phone helplines. The case of the Balearic Islands, Spain
Source: Front Public Health. 2024 Mar 13;12:1270906. doi: 10.3389/fpubh.2024.1270906 (PMC10976841; doi:10.3389/fpubh.2024.1270906)
Supplement: Supplementary file 2 [file Data_Sheet_1.pdf]

CASE NUMBER:

covid19 impact questionnaire

- Date:
- Has the problem(s) you were telephoning about been solved? (tick and write)

  yes

  no

  partly yes partly no

- Type of problem(tick and describe)

  Work related

  Psychological

  Economic

  Housing

  Other

- Can you explain a little more about what this or these problems were?

  Total number of persons currently living at home

  Number of individuals < 18 years old

  Number of individuals 18-65 years old

  Number of individuals > 65 years old

- Employment status of persons aged 16-65 currently living at home (how many in each situation):

  Working

  Unemployed

  ERTO

Other (describe)

- Square metres of the house:

- Number of rooms:

- Access of the house to outside spaces:

    Balcony:

    Terrace (individual or communal):

    Garden:

- Do you have internet access?

    Yes, on the mobile

    Yes, at home

    No

- Finally, I have to ask you a few demographic questions:

    Gender:

    Age:

    Where do you live? (Municipality):

    In which neighborhood or nucleus?(Only if you live in Palma, Calvia, Manacor, Felanitx, Pollença, Alcúdia, Eivissa vila, Sant Antoni, Maó or Ciutadella):

    What is your address? (street and number)

    Where were you born?

        Balearic Islands

        Another autonomous community

        Another country (which one?)
