# Supplementary figures and images for: Assessing the social impacts of the COVID-19 crisis using phone helplines. The case of the Balearic Islands, Spain
Source: Front Public Health. 2024 Mar 13;12:1270906. doi: 10.3389/fpubh.2024.1270906 (PMC10976841; doi:10.3389/fpubh.2024.1270906)

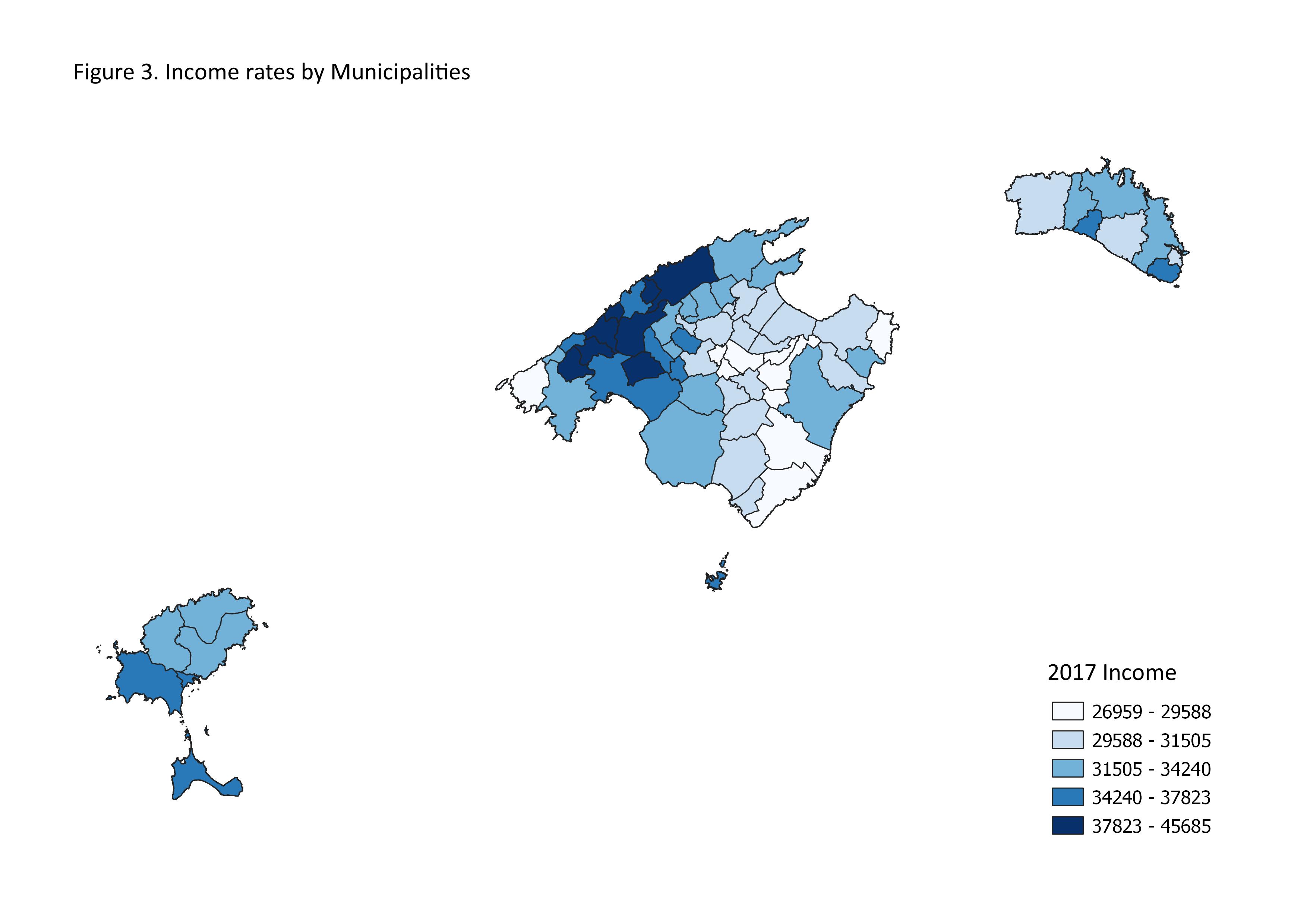

Supplement: Supplementary file 3 [file Image_1.JPEG]

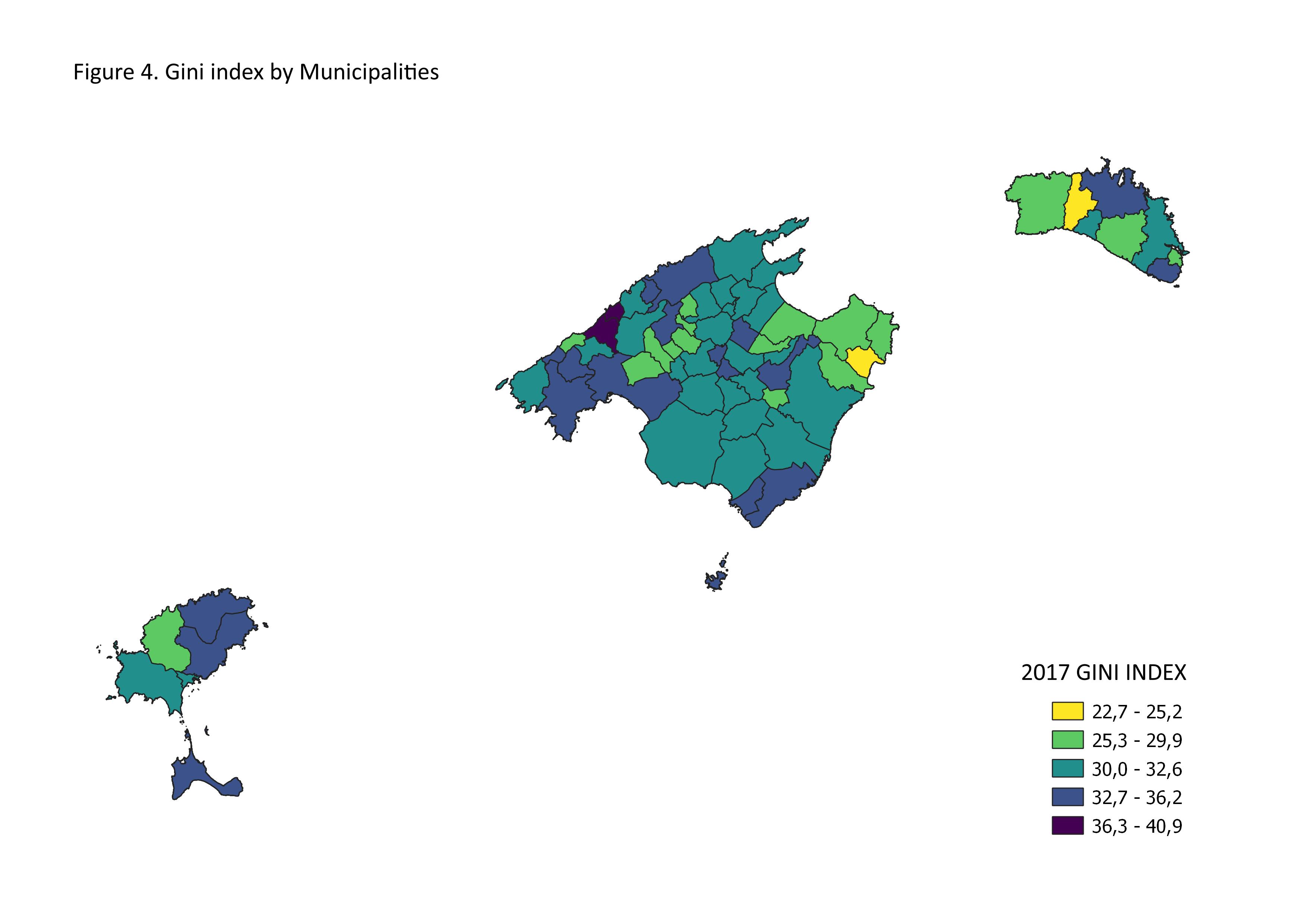

Supplement: Supplementary file 4 [file Image_2.JPEG]

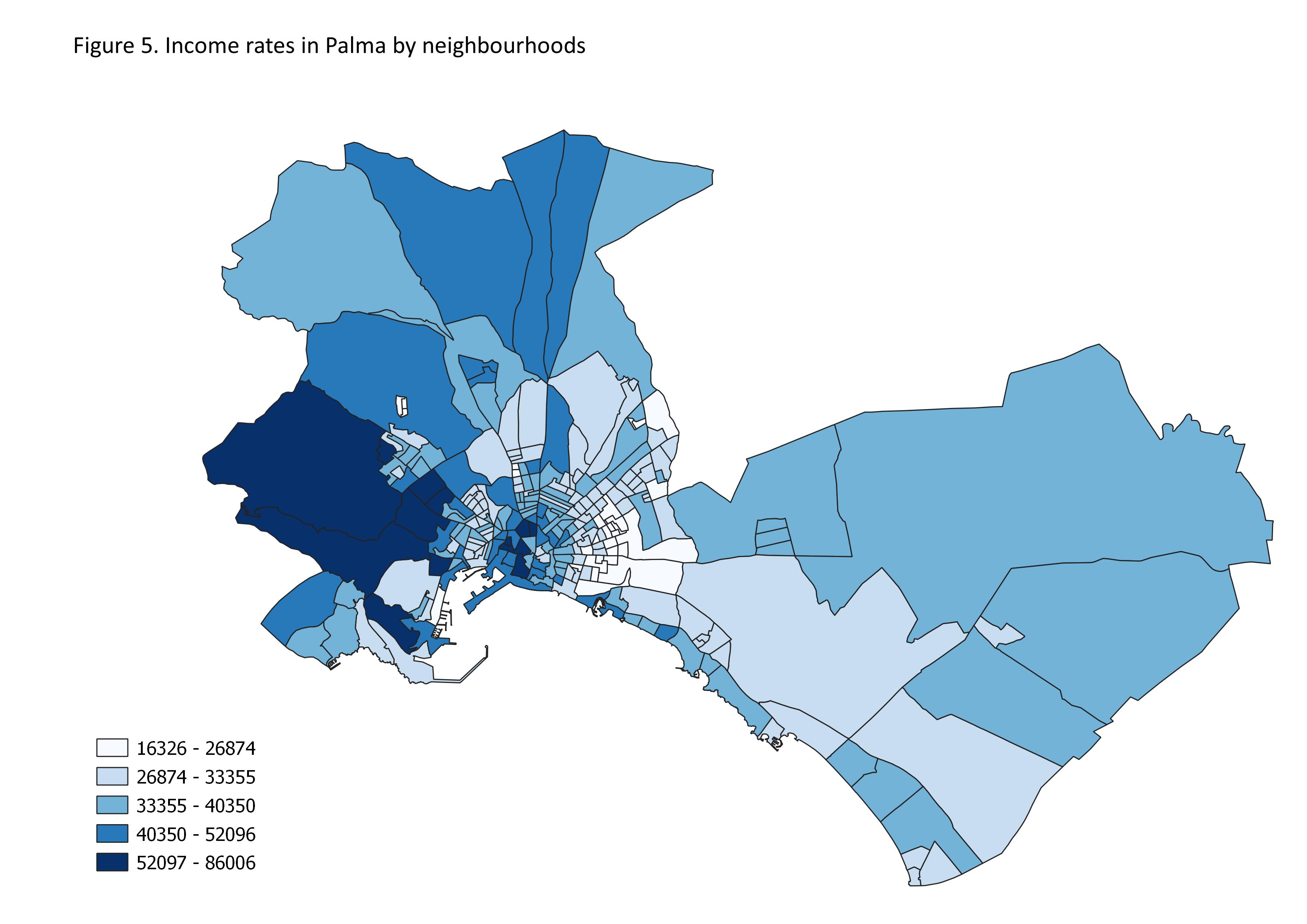

Supplement: Supplementary file 5 [file Image_3.JPEG]

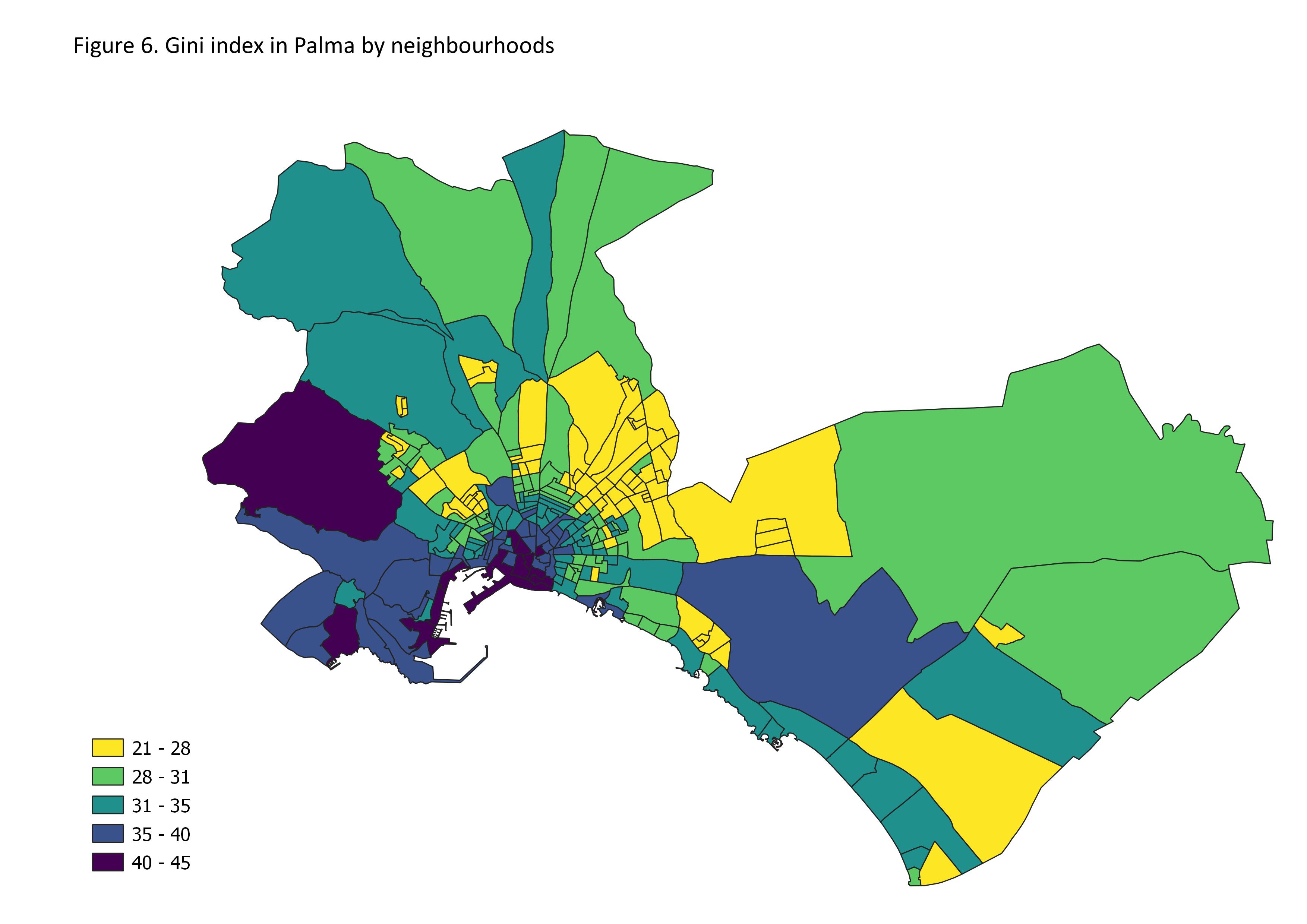

Supplement: Supplementary file 6 [file Image_4.JPEG]
